# Can online peer influencers accelerate HIV testing among young adult males living in Nairobi, Kenya? A study protocol

**DOI:** 10.3389/fpubh.2026.1871838

**Published:** 2026-08-04

**Authors:** Arnold C. Wafula, Evans Amukoye, Kenneth Ngure

**Affiliations:** 1 Department of Environmental Health and Disease Control, School of Public Health, College of Health Sciences, Jomo Kenyatta University of Agriculture and Technology (JKUAT), Nairobi, Kenya; 2 KEMRI Graduate School, School of Public Health, Kenya Medical Research Institute (KEMRI), Centre for Respiratory Disease Research, Nairobi, Kenya

**Keywords:** digital health, HIV prevention, HIV testing, Kenya, young men, peer influencers, randomized controlled trial, WhatsApp

## Abstract

**Background:**

Kenya continues to experience persistent gender disparities in HIV testing. Young men remain substantially less likely than young women to test for HIV, despite expansion of facility based, community based, and HIV self testing services. Although WhatsApp based and peer led interventions have shown promise individually, it remains unclear whether adding online peer influencer engagement to standard HIV communication further increases HIV testing uptake.

**Objective:**

To determine whether adding an online peer influencer led WhatsApp intervention to standard FM radio and WhatsApp HIV messaging increases HIV testing uptake among young adult men aged 18–32 years in Nairobi, Kenya.

**Methods:**

A three-arm, age-stratified randomized controlled trial (1:1:1 allocation) will recruit young adult men aged 18–32 years online in Nairobi. Participants will receive either (i) FM radio HIV messaging, (ii) FM radio plus standard WhatsApp HIV messaging, or (iii) FM radio plus WhatsApp messaging with an online peer influencer intervention. Participants will be followed for 12 weeks. The primary outcome is verified HIV testing uptake, defined as completion of at least one HIV test during follow up and confirmed through participant self report, de-identified facility records, and a Unique Referral Identification Code (URIC). Primary analyses will use generalized linear mixed models under an intention-to-treat framework, with prespecified secondary analyses.

**Discussion:**

This trial addresses an important evidence gap by evaluating the added value of online peer influencers in increasing verified HIV testing uptake among young adult men in Nairobi, beyond standard radio and WhatsApp HIV messaging.

**Conclusion:**

Findings will provide rigorous evidence to inform HIV testing policies and scalable digital behavior change interventions targeting young men in Nairobi and similar urban settings.

**Clinical trial registration:**

https://pactr.samrc.ac.za/Researcher/ManageTrials_v2.aspx, Identifier PACTR202605857599507.

## 1 Introduction

Despite substantial progress in Human immunodeficiency virus (HIV) prevention and treatment, Kenya continues to experience a high HIV burden, with approximately 1.4 million people living with HIV (PLHIV) (1, 2). Additionally, recent increases in HIV incidence and acquired immunodeficiency syndrome (AIDS) related mortality over the past 3 years are of public health concern (2). National surveillance data indicate a steady increase in new HIV infections, from 16,752 cases in 2023 to 19,991 in 2024 and 20,105 in 2025, with approximately 22,000 cases projected for 2026 (2). AIDS related deaths have followed a similar pattern, increasing from 18,473 in 2023 to 20,480 in 2024 and 21,009 in 2025, with approximately 23,000 deaths projected for 2026 (2). Although these trends are likely influenced by multiple factors including the coronavirus disease 2019 (COVID-19) pandemic and changes in funding for HIV/AIDS, among others. They highlight the need for renewed efforts to innovate more cost-efficient HIV testing strategies that maximize cost effectiveness by accelerating early diagnosis and uptake.

While overall national HIV testing uptake has improved, men remain significantly less likely than women to go for an HIV test and to know their HIV status (2). A key driver of Kenya’s increase in HIV incidence is low HIV testing rates. With fewer men than women going for an HIV test to screen for the virus (40). Multiple structural, behavioral, and health system factors contribute to low testing uptake among young men, including HIV related stigma, fear of positive results, concerns about confidentiality, perceived low risk, limited male friendly services, and competing socioeconomic priorities (2, 3). Conventional HIV testing promotion strategies, including facility based outreach and mass media campaigns such as radio, have had limited effectiveness in reaching this population (4). Consequently, internet based, social media interventions are proving effective particularly among young men (1, 5). Although HIV self testing (HIVST), launched in Kenya in (6) to address the stigma associated with facility based HIV testing for men, has expanded access to HIV testing among men, linkage to confirmatory testing and treatment remains suboptimal (2). Fewer than half of HIVST users are successfully linked to care (2, 7). Consequently, innovative interventions that both increase HIV testing and encourage linkage to health facilities remain necessary (1–5).

WhatsApp has emerged as one of the most widely used internet based communication platforms in Kenya, particularly among young adult males living in Nairobi (8–10). According to DataReportal (9) estimates, Kenya has approximately 27.4 million internet users and more than 68 million mobile connections, equivalent to 121% of the population. With about 75% of the Kenyan population under 35 years of age (11, 12), this accounts for what can be described as a youth led digital boom. Making WhatsApp a suitable platform for delivering digital health interventions in Kenya (43).

Nairobi County reports the highest absolute number of new HIV infections of any county in Kenya and has the largest number of HIV Testing Services (HTS) sites nationwide. Combined with high internet connectivity and widespread smartphone use among young adults; these characteristics make Nairobi an appropriate setting for evaluating an innovative social media based intervention to improve HIV testing uptake among young adult men (2, 7, 8).

Chory et al. (53), Ronen et al. (13) and Sheira et al. (14), have demonstrated that in Kenya WhatsApp based peer support interventions are highly acceptable, organically adopted and widely used for communication by young people living with HIV. These studies also demonstrate that WhatsApp can improve HIV related knowledge and behavioral outcomes among young men living with HIV in Kenya. Across sub Saharan Africa, recent studies show an up to 50% increase in HIV testing uptake among men when peer led interventions are used (15, 16). The Owete Trial shows the particular effectiveness of online peer driven interventions at increasing HIV testing uptake among Kenyan men (13). These studies demonstrate that WhatsApp based interventions, peer support programmes and digital health communication can improve young adults’ engagement with HIV services (41). However, it remains unclear whether adding online peer influencer engagement to standard HIV messaging provides additional benefit in increasing verified HIV testing uptake among young adult men in Nairobi, Kenya. This question forms the basis of this randomized controlled trial (RCT).

Although WhatsApp based interventions and peer led approaches have independently demonstrated effectiveness in improving HIV related outcomes, evidence evaluating the additional benefit of adding online peer influencer engagement to standard radio and social media based, HIV testing interventions for young men remains limited. This study addresses that important evidence gap by determining whether adding an online peer influencer component to standard FM radio and WhatsApp, HIV messaging, results in greater HIV testing uptake among young adult men in Nairobi.

## 2 Methods

### 2.1 Study design

This study is a three arm, parallel group, age stratified; randomized controlled trial (RCT) with equal allocation (1:1:1) will be conducted over a 12 week period. The trial compares the effectiveness of three different HIV testing uptake promotion strategies of increasing intensity: Arm 1 (active control), Arm 2 (comparator), or Arm 3 (intervention). Participants will be followed for 12 weeks, with outcome assessments at baseline (0) and weeks 2, 6, 10, and 12 using a repeated measures framework (17, 18, 56).

Consistent with the parallel group design framework established by Schulz et al. (18), the three different study arms (WhatsApp groups) will each concurrently receive different interventions promoting HIV testing uptake during the 12 week study period.

### 2.2 Trial registration

The trial was registered with the Pan African Clinical Trials Registry (PACTR) (Trial ID: PACTR202605857599507) prior to participant recruitment to ensure transparency and reduce the risk of selective reporting. The protocol was developed in accordance with the Standard Protocol Items: Recommendations for Interventional Trials (SPIRIT) 2013 Statement (19). The completed trial will be reported in accordance with the Consolidated Standards of Reporting Trials (18, 60). Ethical approval was obtained from the Kenya Medical Research Institute (KEMRI) Scientific and Ethics Review Unit (SERU), and research authorization was granted by the National Commission for Science, Technology and Innovation (NACOSTI) before participant recruitment.

### 2.3 Rationale for not conducting a pilot study

Operating without an independent pilot phase, the operational architecture of this RCT is directly modeled upon extensive research of highly similar trials demonstrating that both full digital interventions and online peer influencer frameworks effectively increase HIV testing uptake among young adult males in urban East Africa (15, 16).

### 2.4 Study setting

This study will be conducted in Nairobi County, Kenya, using a hybrid online-to-facility design that integrates digital HIV testing demand generation with facility based HIV testing and linkage to care.

#### 2.4.1 Geographic context

The study will be conducted in Nairobi, Kenya’s capital and largest urban centre. Nairobi has a large population of young adults, widespread smartphone ownership, high internet connectivity, and extensive use of social media platforms, particularly WhatsApp. Making it an appropriate setting for evaluating digitally delivered HIV testing interventions.

Nairobi consistently reports the highest absolute number of new HIV infections among Kenya’s counties and remains a priority area for HIV prevention and testing interventions (2). Although HIV prevention and testing services are widely available, gender disparities persist, with young men demonstrating lower HIV testing uptake and lower linkage to care than women, contributing to disproportionately higher AIDS related mortality rates among men (2).

Nairobi has an extensive network of Ministry of Health accredited HIV Testing Services (HTS) facilities and Comprehensive Care Clinics (CCCs), providing appropriate infrastructure for HIV testing, outcome verification, and linkage to care during the trial (51).

#### 2.4.2 Digital context

Nairobi has high levels of internet connectivity, smartphone ownership, and social media use, particularly among young adults. WhatsApp is one of the most widely used messaging platforms in Kenya, providing a scalable and acceptable platform for peer driven health communication and digital intervention delivery (8).

#### 2.4.3 Partner facility

The study will partner with a Ministry of Health-accredited Level 2 community health facility located in Kibra Sub-county, Nairobi. The facility provides 24-h outpatient services, HIV Testing Services (HTS), a Comprehensive Care Clinic (CCC), tuberculosis (TB) treatment, maternity services, and minor surgical procedures. The facility is registered on the Ministry of Health Master Facility List (MFL) and will serve as the primary site for HIV testing verification and linkage to care during the trial (20).

#### 2.4.4 Hybrid online-to-facility model

The study employs a hybrid online-to-facility intervention model in which HIV testing demand is generated on WhatsApp, while HIV testing, verification, and linkage to care occur through the participating partner HTS facility (1, 21). This model seeks to address the persistent gap in linkage to care by encouraging participants to access confirmatory HIV testing and HIV care through the partner HTS facility (2, 7). Participants assigned to the intervention arm will be encouraged by trained online peer influencers to attend the partner HTS facility for HIV testing and linkage to care where appropriate.

The hybrid model combines:

- *Digital delivery:* Recruitment, screening, informed consent, enrolment, randomization, intervention delivery, follow up, and participant engagement via WhatsApp
- *Facility based services:* HIV testing, confirmatory testing where required, referral code redemption, outcome verification, and linkage to care

### 2.5 Study population

Young adult men were selected as the target population because they consistently demonstrate lower HIV testing uptake, delayed diagnosis, lower engagement across the HIV care cascade, and higher AIDS related mortality than women despite representing a smaller proportion of people living with HIV in Kenya (1, 2).

#### 2.5.1 Target population

The target population comprises all young adult men aged 18–32 years residing in Nairobi County.

#### 2.5.2 Study population

The study population comprises all eligible young adult men aged 18–32 years residing in Nairobi County who meet the eligibility criteria, provide informed consent, and are recruited through the study’s online recruitment strategy.

#### 2.5.3 Sampling frame

The sampling frame consists of eligible members of the partner FM radio station’s Facebook community and affiliated digital platforms who voluntarily respond to the online recruitment campaign.

### 2.6 Recruitment

Recruitment will be conducted entirely online. Study advertisements will be broadcast through the partner FM radio station and disseminated via its Facebook page and other approved digital platforms. Interested individuals will access an online eligibility screening questionnaire through a study specific Uniform Resource Locator (URL) or Quick Response (QR) code.

Eligible participants will complete electronic informed consent followed by the baseline questionnaire. Recruitment, screening, consent, enrolment, and follow up will all be conducted online. No face-to-face or community based recruitment will occur.

Recruitment channels include:

- Partner FM radio broadcasts
- Facebook recruitment
- Dissemination through partner organizations and community networks

### 2.7 Eligibility criteria

#### 2.7.1 Inclusion criteria

Participants must:

- Be male
- Be aged 18–32 years
- Reside in Nairobi County
- Own or have regular access to a smartphone with WhatsApp
- provide electronic informed consent

#### 2.7.2 Exclusion criteria

Participants will be excluded if they:

- Are currently receiving antiretroviral therapy
- Are participating in another HIV testing intervention
- Lack access to WhatsApp
- Are unable or unwilling to provide informed consent

### 2.8 Sample size determination

The primary outcome of this trial is HIV testing uptake, defined as completion of at least one HIV test during the study period. Because the primary outcome is binary and reported as a population proportion, the sample size was estimated using Cochran’s formula for proportions (22).


$$n0=(Z^{2}\cdot p\cdot q)/e^{2}$$

Where *Z* represents the critical value for a 95% confidence level (1.96), *e* denotes the acceptable margin of error (0.05), and p represents the estimated baseline proportion of HIV testing uptake. Given that the true HIV testing prevalence within this online male demographic was undetermined, *p* was conservatively set at 0.50 (*q* = 1 − *p* = 0.50) to assume maximum population variance and yield the most conservative sample size baseline. This primary calculation established a baseline requirement of 384 participants. To account for anticipated digital non-response and loss to follow-up, an approximate 22% attrition adjustment was applied (inflating the sample via *n* = *n*₀/0.82), resulting in a final cleared sample size of 468 participants allocated equally across the three study arms in a 1:1:1 ratio (156 participants per arm). Within each arm, participants will be stratified by age group prior to simple randomization to preserve developmental balance.

To validate this allocation against the primary objective, a supplementary power assessment was conducted using the standard formula for comparing two independent proportions:


$$n=[{\left(Z\_\{\alpha /2\}+Z\_\beta \right)}^{2}\cdot (p₁(1-p₁)+p₂(1-p₂))]/{\left(p₁-p₂\right)}^{2}$$

Where *α* is fixed at a two-sided significance level of 0.05 (*Z*_{*α*/2} = 1.96) and statistical power is fixed at 80% (*Z*_*β* = 0.84). Based on evidence highlighting depressed health seeking behaviors among young men in Kenya alongside historical peer led intervention data demonstrating up to 50% relative increases in testing uptake within sub-Saharan Africa, the calculation assumed an expected HIV testing uptake rate of 20% (*p*₁) in the active control arm and 40% (*p*₂) in the intervention arms (15, 16). This establishes an estimated absolute effect size of 20 percentage points.

Processing these assumptions confirms that an allocation of 156 participants per arm is statistically sufficient to detect absolute differences of approximately 20 percentage points between any single intervention arm and the active control group. While generalized linear mixed models (GLMMs) will be implemented for the longitudinal analysis of repeated outcome measurements, these serve purely as analytical frameworks rather than sample size estimation methods (17, 23). The trial is explicitly not powered to identify subtle variations within participant subgroups or to support definitive head-to-head hypothesis testing between the two intervention arms.

The study is powered to detect large intervention effects of approximately 20 percentage points in HIV testing uptake between study arms at a two-sided significance level of 0.05 and 80% statistical power. Subgroup analyses will be considered exploratory and interpreted cautiously (42).

### 2.9 Stratification, randomization and allocation

Following consent and baseline assessment, participants will be stratified into three prespecified age groups:

- *Stratum A*: 18–22 years (Young Gen Z)
- *Stratum B*: 23–27 years (Older Gen Z)
- *Stratum C*: 28–32 years (Young Millennials)

This stratification approach is informed by evidence demonstrating age related differences in HIV testing uptake and health seeking behaviors among young men (24), as well as variation in peer influence within social networks (25).

Each stratum will include 156 participants, with equal allocation across study arms (52 participants per arm per stratum), yielding a total sample size of 468 participants.

Within each stratum, participants will be randomly assigned in a 1:1:1 ratio using computer generated block randomization with a fixed block size of six. Randomization sequences will be prepared independently before recruitment by an independent randomization officer not involved in enrolment.

**Figure 1:**
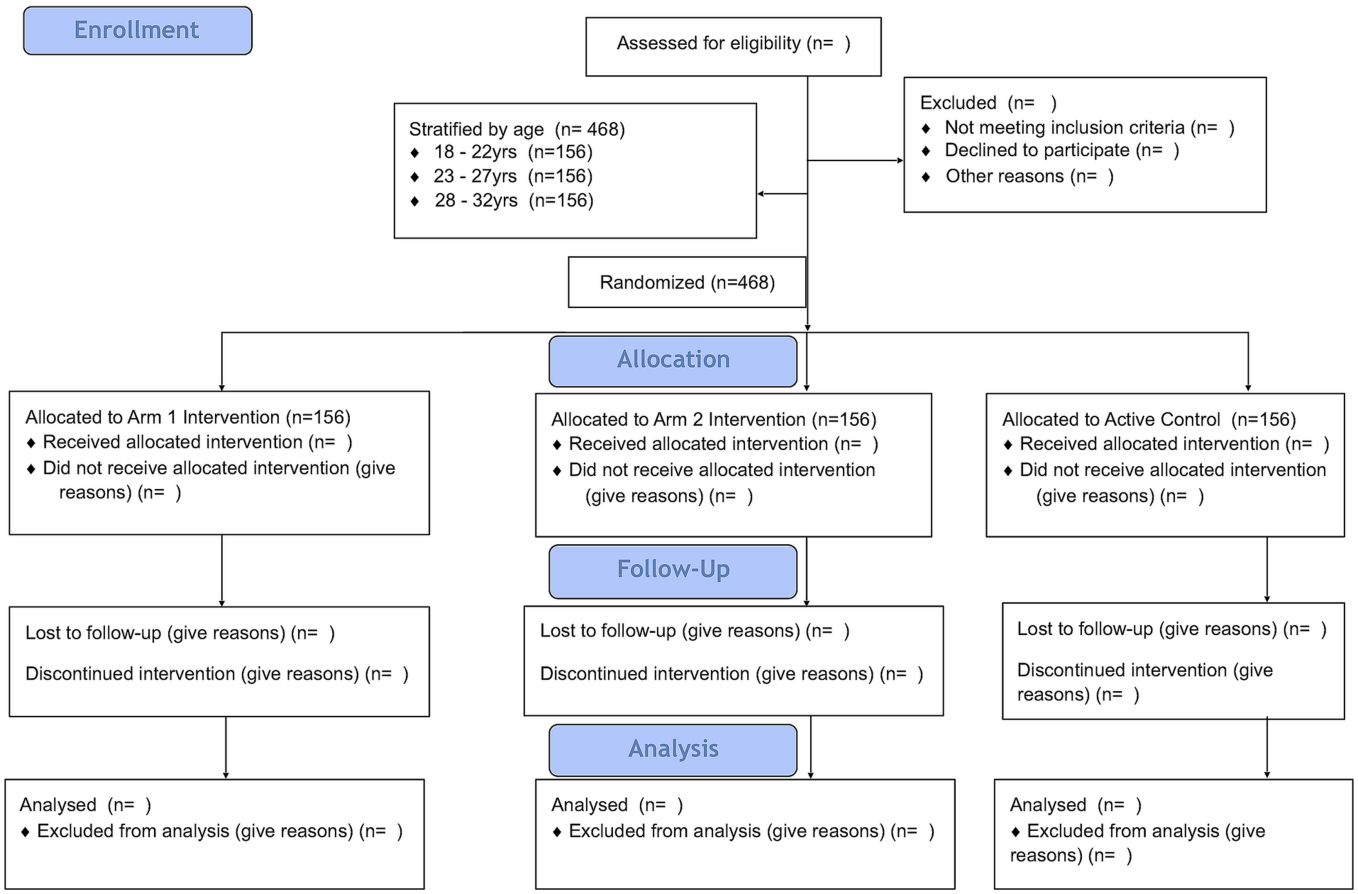
CONSORT flow diagram showing participant progression through screening, allocation, follow-up, and analysis Adapted from CONSORT, 2010 (18, 39). License: Open Access/Public Domain. Source: consort-statement.org.

**Figure 2:**
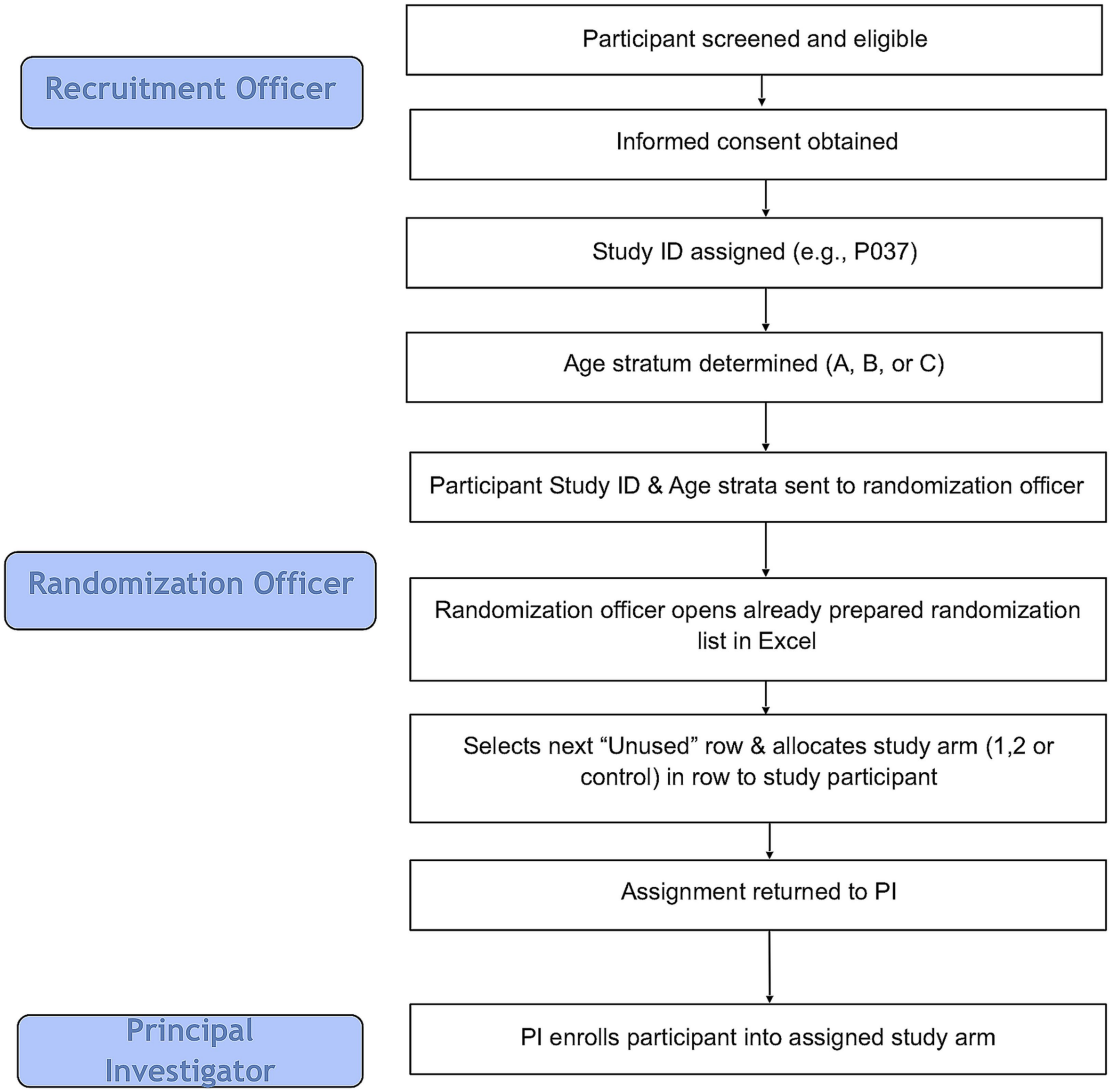
Randomization and allocation workflow diagram. Operational workflow for participant randomization and allocation using stratified block randomization. Original diagram created by Wafula AC.

Allocation concealment will be maintained using centralized password-protected computerized randomization. Recruitment staff will remain unaware of future allocations until assignment has occurred (see Figures 1, 2).

### 2.10 Intervention Arms

Participants will be randomly allocated to one of three intervention arms of increasing intervention intensity.

*Arm 1 (active control)*: participants will receive routine HIV promotion messages broadcast through the partner FM radio station.

*Arm 2 (standard digital messaging)*: participants will receive the FM radio intervention together with standardized HIV related WhatsApp messages delivered through closed WhatsApp study group.

*Arm 3 (peer influencer intervention)*: participants will receive the FM radio intervention, standardized WhatsApp messaging, and additional engagement from trained online peer influencers who will facilitate discussions, share structured HIV testing messages, provide behavioral prompts and reminders, answer general questions, and encourage HIV testing at the partner HIV Testing Services (HTS) facility.

Interventions will be delivered over a 12-week period.

### 2.11 Online peer influencer intervention

A total of 15 online peer influencers (five per age stratum) will be identified using social network analysis (SNA) methods. This approach enables objective selection of individuals with high influence within digital networks, improving intervention reach and effectiveness compared to convenience-based peer educator models (25).

Peer influencers will complete standardized virtual training covering HIV communication, ethical conduct, participant engagement, confidentiality, and referral procedures. Throughout the intervention they will receive ongoing supervision, weekly monitoring, and fidelity assessments.

Peer influencers will facilitate WhatsApp discussions, encourage HIV testing, distribute referral information, and support participant engagement. They will not participate in recruitment, randomization, outcome assessment, or data analysis (44).

### 2.12 Contamination prevention

To minimize contamination, each study arm will operate within separate closed WhatsApp groups. Participants will have no interaction across study arms, and peer influencers will communicate only with participants assigned to their respective intervention group.

Potential contamination will be assessed during process evaluation (26).

### 2.13 Masking

Because of the behavioral nature of the intervention, participants and peer influencers cannot be blinded.

Outcome verification will be conducted independently wherever feasible, while data analysts will analyze masked treatment codes until completion of the primary analyses.

**Figure 3:**
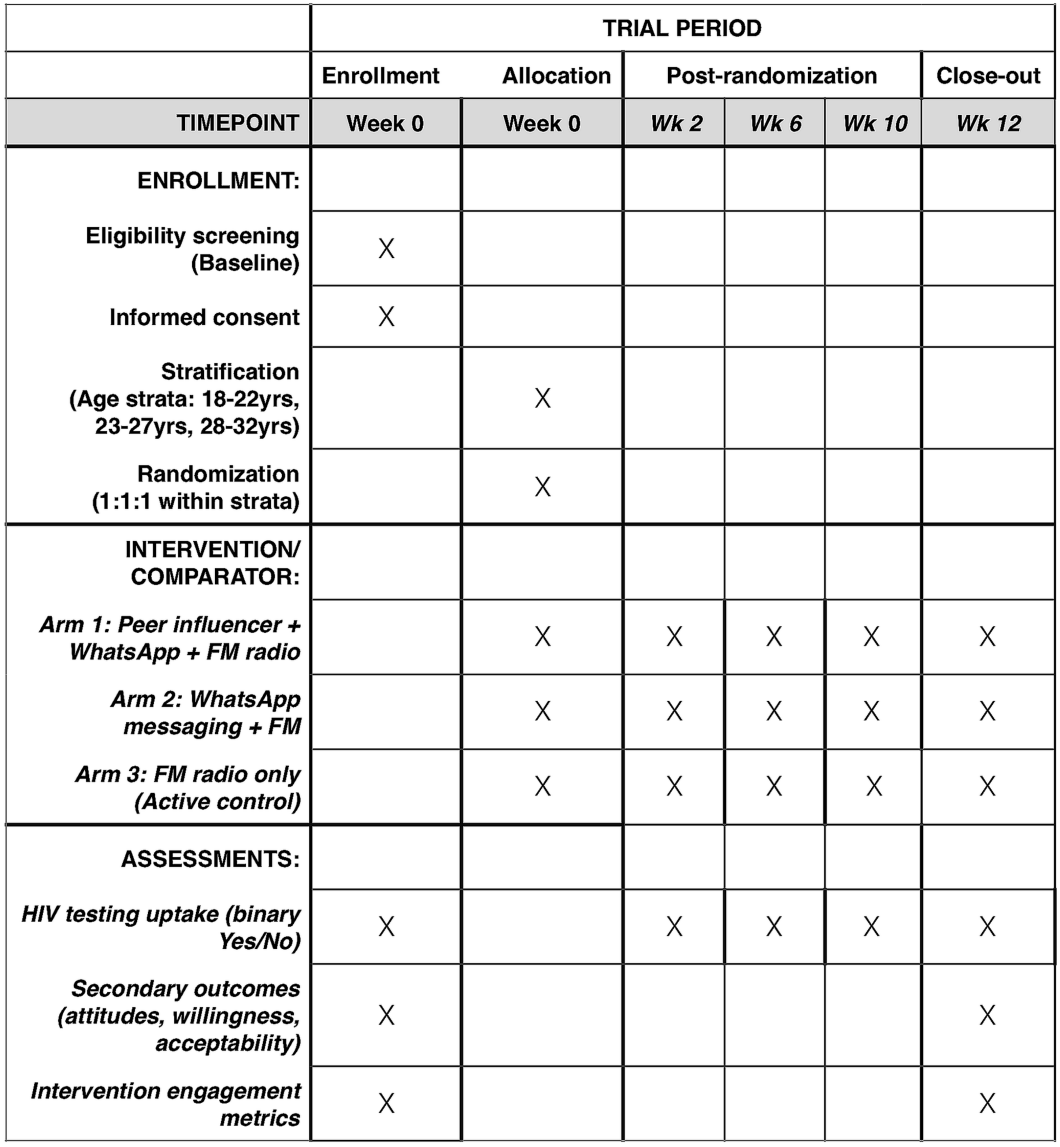
SPIRIT schedule of enrollment, allocation, interventions and assessments. Adapted Chan et al., SPIRIT 2013 Statement (19). License: Open Access/Creative Commons CC BY-NC-ND. Source: spirit-statement.org.

This partial masking approach is consistent with best practices for non pharmacologic trials and is intended to reduce ascertainment and analytical bias (27) (see Figure 3).

### 2.14 Compensation

Peer influencers will receive reimbursement for reasonable internet data and communication costs incurred during the study. No payment will be provided for participation time, and compensation will not constitute an inducement to participate. This compensation is intended to support participation without constituting undue inducement, in line with ethical research guidelines (28).

### 2.15 Outcome measures

#### 2.15.1 Primary outcome

The primary outcome is HIV testing uptake, defined as completion of at least one HIV test during the 12 week follow up period after randomization, irrespective of HIV test result.

#### 2.15.2 Secondary outcomes

Secondary outcomes include:

- Time to first HIV test
- Repeated HIV testing uptake over time
- Differences between age strata
- Participant engagement
- Intervention acceptability
- Linkage to HIV care
- Cost effectiveness

Qualitative outcomes will be explored through focus group discussions and key informant interviews analyzed using thematic analysis.

### 2.16 Outcome verification

PEach participant will receive a Unique Referral Identification Code (URIC) for enrolment. The URIC is a unique study generated identifier that anonymously links participant self reports with partner health facility records without recording personal identifiers in the research database.

Participants will present their URIC whenever attending the partner HTS facility for HIV testing. Facility staff will record the URIC together with confirmation that HIV testing was completed.

HIV testing uptake will be verified using three complementary data sources:

- Participant self report;
- Anonymized partner facility HIV testing records; and
- URIC redemption.

A participant will be classified as having completed HIV testing only after facility verification confirms test completion. This hybrid verification model minimizes outcome misclassification, reduces recall bias, strengthens internal validity, and preserves participant confidentiality (29). This approach improves validity by reducing reliance on self report alone and is consistent with best practices in behavioral intervention trials (29).

### 2.17 Data collection

Data for this study will be collected using a fully digital, longitudinal approach aligned with the hybrid trial design, integrating repeated measures, online platform derived engagement data, and anonymized facility based verification (52, 57).

Data collection procedures are prespecified in the study Data Management Plan (DMP; DMPTool Registry No.10.48321D1D8C7728A), ensuring transparency and reproducibility (30). Data will be collected at baseline (Week 0) and during follow up assessments at Weeks 2, 6, 10, and 12 using standardized electronic data collection instruments.

Baseline data will include demographic characteristics, socioeconomic characteristics, HIV testing history, HIV related knowledge, digital media use, and other variables relevant to the study objectives.

Follow up assessments will capture intervention exposure, participant engagement, HIV testing behavior since the previous assessment, linkage to care, adverse events, and protocol deviations.

All questionnaires will be completed electronically through secure online platforms using standardized instruments across all study arms.

Intervention engagement will be monitored using WhatsApp platform metrics, including message delivery, read status, participant responses, and interaction frequency to assess intervention fidelity.

#### 2.17.1 Survey instrument

The primary outcome will be measured using standardized electronic questionnaires administered at each follow up assessment.

Participants will be asked:

*“At any time during the past 4 weeks, have you had an HIV test?”*

Responses will be recorded as Yes or No.

#### 2.17.2 Data collection time points

Data will be collected at five predefined study time points:

- Baseline (Week 0; preintervention)
- Week 2 (early exposure)
- Week 6 (midline)
- Week 10 (late exposure)
- Week 12 (endline)

This repeated measures structure enables longitudinal analysis across study arms (23).

#### 2.17.3 Baseline assessment

Baseline assessment will collect:

- Age
- Education
- Employment status
- Previous HIV testing history
- HIV-related knowledge
- Digital access and WhatsApp use
- Behavioral risk characteristics

#### 2.17.4 Intervention engagement

Intervention engagement will be monitored using WhatsApp interaction metrics, including message delivery, read status, participant responses, and participation frequency. These indicators will be used to assess intervention fidelity (31).

#### 2.17.5 Retention and attrition management

Retention strategies will include automated reminders, responsive participant communication, and continuous engagement via study channels (32).

Attrition will be monitored weekly. Where attrition exceeds predefined thresholds, additional participant follow-up strategies will be implemented.

### 2.18 Data management

All data will be collected and stored electronically using secure systems, including encrypted, password protected platforms. WhatsApp identifiers will be replaced with unique study IDs, and only de-identified datasets will be used for analysis. Access to identifiable data will be restricted to authorized personnel. Daily encrypted backups will be maintained.

Data management procedures are specified in the study Data Management Plan (DMPTool Registry No. 10.48321D1D8C7728A).

Study data will be collected and stored electronically using encrypted password protected systems.

Participants will be identified only by unique study identification numbers. Personally identifiable information will be stored separately from research data. Outcome verification data obtained from partner health facilities will be linked using the URIC without recording participant names within the analytical dataset.

Access to identifiable information will be restricted to authorized study personnel. Encrypted backups will be performed regularly.

Data management procedures comply with international security standards [ISO/IEC 27001; (33)].

#### 2.18.1 Data protection

All procedures comply with the Kenya Data Protection Act (2019) and General Data Protection Regulation (GDPR) (34) aligned principles of confidentiality, integrity, and lawful processing.

Participants will be identified only by unique study codes. Research and facility data will be stored separately, ensuring full anonymization prior to analysis.

#### 2.18.2 Data quality

Data quality will be maintained through electronic validation checks, routine consistency reviews, periodic audits, and standardized operating procedures. Data quality monitoring will follow International Council for Harmonisation Good Clinical Practice (35) guidelines.

##### 2.18.2.1 Documentation and metadata (FAIR principles)

Study documentation, metadata, and analysis scripts will comply with FAIR (Findable, Accessible, Interoperable, and Reusable) data principles (30).

#### 2.18.3 Data analysis plan

Data analysis will follow an intention-to-treat (ITT) approach (36).

Analytical methods will include:

- Descriptive statistics
- Chi-square tests (55)
- McNemar’s and Cochran’s *Q* tests (22, 58)
- Logistic regression (37)
- Thematic qualitative analysis
- Cost-effectiveness analysis (38)

#### 2.18.4 Data sharing

De-identified datasets and analysis scripts will be preserved and shared in accordance with FAIR principles. Data will be deposited at the Kenya Medical Research Institute (KEMRI) and or Jomo Kenyatta University of Agriculture and Technology (JKUAT) Institutional Repositories and retained for a minimum of 10 years.

#### 2.18.5 Responsibility

The Principal Investigator (PI) will have overall responsibility for data governance and oversight (49).

### 2.19 Statistical analysis

All analyses will follow a prespecified Statistical Analysis Plan (SAP) deposited with the PACTR registry. Analyses will be conducted under an intention-to-treat framework to preserve randomization integrity (36).

A two sided significance level of 0.05 will be used throughout, with 95% confidence intervals reported (48).

#### 2.19.1 Primary analysis

The primary outcome will be analyzed using generalized linear mixed effects models (GLMMs) with a link to compare HIV testing uptake between study arms over time while accounting for within participant correlation.

Fixed effects will include:

- Study arm
- Follow up time
- Arm × time interaction

Random intercepts will account for repeated observations within participants.

#### 2.19.2 Secondary analyses

Secondary analyses will include:

- Descriptive statistics
- Chi-square tests
- Cochran’s *Q* tests
- McNemar’s tests
- Multivariable logistic regression
- Age-stratified subgroup analyses
- Cost-effectiveness analysis

#### 2.19.3 Missing data

Missing outcome data will be examined and, where appropriate, addressed using multiple imputation. Sensitivity analyses will assess the robustness of study findings (45).

#### 2.19.4 Software

Analyses will be conducted using R, JAMOVI, or Stata.

## 3 Discussion

This protocol describes a three-arm, age-stratified randomized controlled trial evaluating whether adding an online peer influencer led WhatsApp intervention to standard FM radio and WhatsApp HIV messaging increases HIV testing uptake among young adult men in Nairobi, Kenya. The study combines digital demand generation with facility based HIV testing verification using a Unique Referral Identification Code (URIC), providing an objective approach to outcome measurement while preserving participant confidentiality.

The trial addresses an important evidence gap by evaluating the additional value of online peer influencer engagement beyond existing HIV communication strategies (46). Methodological strengths include randomized allocation, age stratification, repeated outcome assessments, intention-to-treat analysis, objective verification of HIV testing uptake, and implementation in a real world urban setting (50).

Several limitations should be acknowledged. Blinding of participants and peer influencers is not feasible because of the behavioral nature of the intervention and may introduce performance bias (27). In addition, recruitment through online platforms may limit participation among individuals with limited internet access, potentially affecting generalizability (54). Although facility based verification strengthens outcome validity, HIV tests completed outside participating facilities may not be captured despite participant self report (18, 59).

If effective, this intervention could provide a scalable, low cost digital strategy for increasing HIV testing uptake among young men and inform national HIV prevention programmes in Kenya and other comparable settings (2, 7, 24, 47).
